# Supplementary material for: Multiomics Molecular Research into the Recalcitrant and Orphan Quercus ilex Tree Species: Why, What for, and How
Source: Int J Mol Sci. 2022 Sep 1;23(17):9980. doi: 10.3390/ijms23179980 (PMC9456323; doi:10.3390/ijms23179980)
Supplement: Supplementary file 1 [file ijms-23-09980-s001.zip › Figure S1 Maldonado-Alconada et al.pdf]

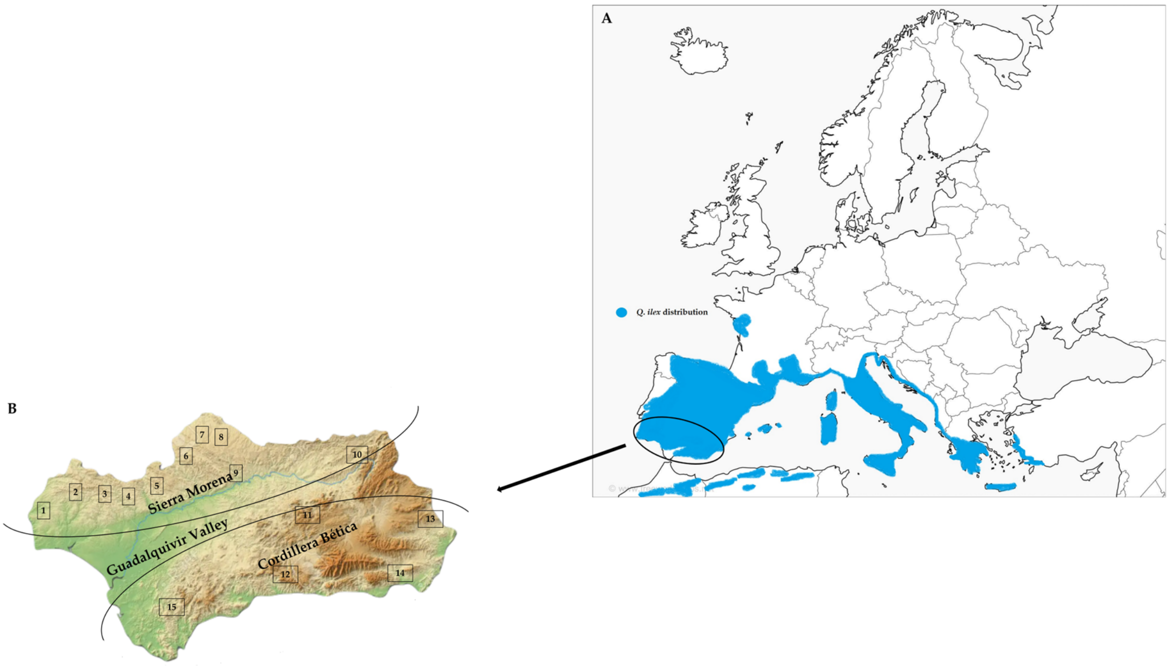

Figure S1. (A) Quercus ilex distribution in the Mediterranean forest and the agrosylvopastoral ecosystem "dehesa" (Spain) adapted from [169]. (B) Map of Andalusia (inset showing position of Andalusia in Europe) showing the following sampling locations used by the authors' research group: 1. Paymogo (Huelva); 2. Aroche (Huelva); 3. Corteconcepción (Huelva); 4. Almadén de la Plata (Sevilla); 5. Cazalla de la Sierra (Sevilla); 6. Fuente Obejuna (Córdoba); 7. Hinojosa del Duque (Córdoba); 8. El Viso (Córdoba); 9. Cerro Muriano (Córdoba); 10. Hornos de Segura (Jaén); 11. Valdepeñas de Jaén (Jaén); 12. Arenas del Rey (Granada); 13. Sierra María (Almería); 14. Sierra Alhamilla (Almería); 15. Benamahoma (Cádiz) (map of Andalusia adapted from https://es.123rf.com/photo);.
